# Achilles tendon allograft implantation for anterior axillary fold reconstruction in Poland syndrome

**DOI:** 10.1016/j.jpra.2026.06.009

**Published:** 2026-06-26

**Authors:** Yaiel G. Rodríguez-Avilés, Felix M. Rivera-Troia, Hiram E. Luigi-Martinez, Joseph Salem-Hernández, Eduardo González, Carlos J. Pérez-López, Gustavo E. Bello

**Affiliations:** a Department of Surgery, Saint Luke’s Episcopal Medical Center, Ponce, Puerto Rico, 00717, USA; b Department of Surgery, Section of Orthopedic Surgery, Ponce Health Sciences University, School of Medicine, Ponce, Puerto Rico, 00717, USA; c Department of Surgery, Section of Plastic Surgery, Ponce Health Sciences University, School of Medicine, Ponce, Puerto Rico, 00717, USA

**Keywords:** Poland syndrome, Anterior axillary fold, Achilles tendon allograft, Chest wall reconstruction, Tendon-osseous repair, Pectoralis major

## Abstract

**Background:**

Poland Syndrome (PS) is a rare congenital condition characterized by unilateral pectoralis major hypoplasia or aplasia, often with associated anterior axillary fold deficiency. Conventional reconstructive options carry inherent limitations including donor-site morbidity and unpredictable volume retention. This report describes the first use of an Achilles tendon allograft to reconstruct the anterior axillary fold in a PS patient with prior silicone implant placement.

**Case:**

A 48-year-old male presented with persistent anterior axillary fold deficiency following prior left pectoralis major reconstruction with a silicone implant. Reconstruction was performed using an Achilles tendon allograft secured to the proximal humerus via suture anchors and to the implant capsule through a tendon-osseous repair technique adapted from pectoralis major rupture surgery.

**Findings:**

At four-year follow-up, the patient maintained full shoulder range of motion, durable anterior axillary fold contour, and satisfactory cosmetic outcome, with no graft failure, implant displacement, or animation deformity.

**Conclusion:**

Achilles tendon allograft implantation offers a novel, less invasive reconstructive option for anterior axillary fold restoration in PS, avoiding donor-site morbidity while providing durable structural support.

## Introduction

Poland Syndrome (PS) is defined by congenital unilateral hypoplasia or aplasia of the sternocostal pectoralis major, often with ipsilateral deficiencies of the pectoralis minor, costal cartilage, breast, or upper limb.^1^ Its prevalence is approximately 1 in 20,000–30,000 live births, with a male and right-sided predominance.^2^ Anterior axillary fold involvement significantly affects chest wall symmetry and causes considerable psychosocial burden, particularly during adolescence and early adulthood.

Among the spectrum of PS deformities, anterior axillary fold deficiency is one of the most challenging to address. The fold is a three-dimensional structure defined by the inferior border of the pectoralis major as it converges toward its humeral insertion; its absence creates a visible contour asymmetry that is difficult to recreate with volume-based techniques alone. Established reconstructive strategies include the latissimus dorsi (LD) flap, gracilis and anterolateral thigh (ALT) flaps, fat grafting, and custom or silicone implants.^3^, ^4^, ^5^, ^6^ Each carries limitations: LD flaps involve donor-site morbidity and potential shoulder weakness, fat grafting yields unpredictable volume retention, and silicone implants (SI) alone fail to reconstitute the anterior axillary fold contour.^5^^,^^6^

Achilles tendon allograft has not been previously reported for anterior axillary fold reconstruction. Its tubular architecture, structural durability, and favorable handling properties closely mimic the natural fold contour, while eliminating donor-site morbidity and providing lasting volume without resorption. We describe the first documented use of this technique in a PS patient with prior SI placement, adapting tendon-osseous repair principles typically employed in pectoralis major rupture surgery.^7^^,^^8^

## Case presentation

**Fig. 1:**
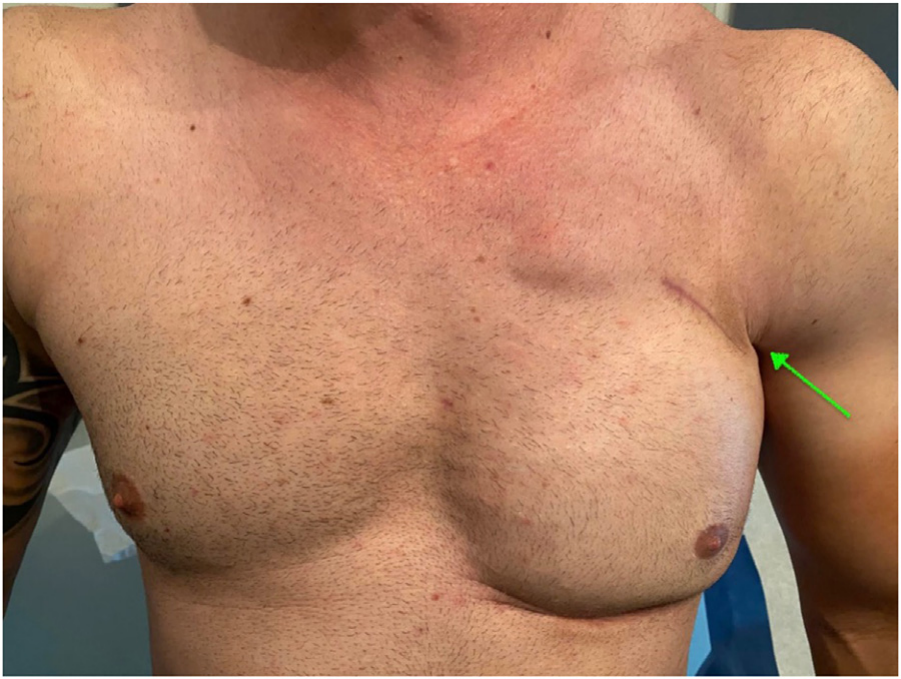
Preoperative photograph demonstrating a prior low-riding silicone implant in situ with complete absence of the anterior axillary fold (green arrow). Written patient consent for publication of this image was obtained.

A 48-year-old male with PS presented seeking cosmetic revision following prior left pectoralis major reconstruction with a SI at an outside institution. He reported no pain, functional limitations, or implant-related symptoms, but was dissatisfied with the persistent anterior axillary fold deformity. Physical examination revealed surgical scars from the prior procedure, a low-riding SI, and complete absence of the anterior axillary fold (Fig. 1). Shoulder range of motion was intact bilaterally. After informed discussion of reconstructive options including LD flap transfer, fat grafting, and allograft reconstruction, the patient elected to proceed with anterior axillary fold restoration using an Achilles tendon allograft, citing preference for avoidance of additional donor-site scars and muscle harvest. Written informed consent was obtained, including consent for allograft use and publication of clinical photographs.

## Surgical technique

The patient was positioned in the beach chair position with appropriate padding and cervical alignment ensured. Intraoperative findings confirmed congenital absence of the pectoralis major, an existing SI, and a deficient axillary contour. A modified deltopectoral approach focused on the distal portion allowed access to the pectoralis major humeral insertion. The cephalic vein was identified and carefully protected throughout. The long head of the biceps tendon served as a landmark for the expected pectoralis footprint, which was curetted to promote biological healing at the graft-bone interface. A commercially available, aseptically processed and fresh-frozen Achilles tendon allograft was obtained from LifeLink Tissue Bank (Tampa, FL, USA), an AATB-accredited tissue bank. The graft was thawed per the supplier protocol in room-temperature normal saline for approximately 40 min and prepared on the back table. After trimming, the tendon graft measured approximately 15 cm in length, with the calcaneal bone strut removed prior to implantation. The graft was kept moist with saline-soaked gauze until implantation. Two double-loaded suture anchors were used to securely fix the proximal end of the Achilles tendon allograft to the humerus.

**Fig. 2:**
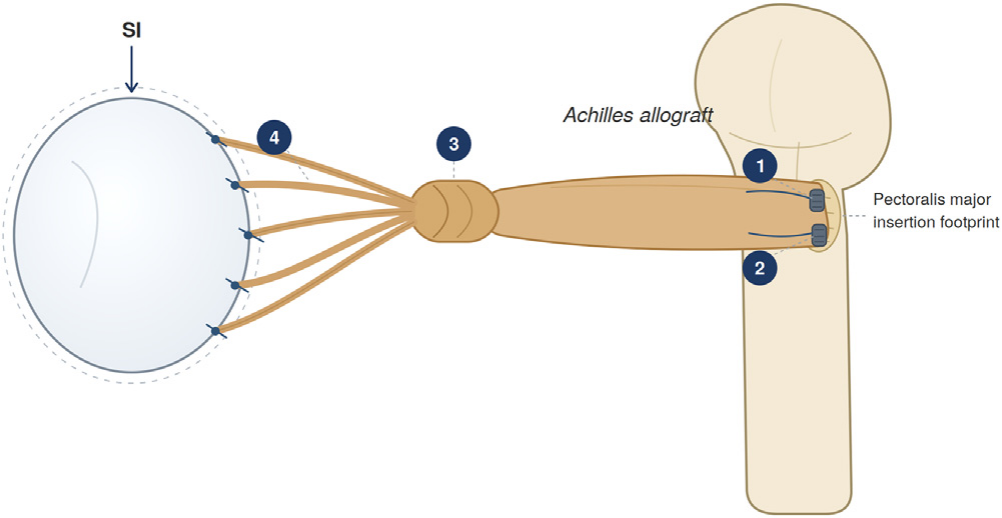
Schematic of the reconstruction. (1) pectoralis major insertion footprint identified and curetted at the humeral attachment; (2) double-loaded suture anchors securing the proximal end of the Achilles allograft to the humerus; (3) allograft tubularized at the axillary crease to augment fold volume; (4) medial allograft end fanned out and sutured to the existing silicone implant capsule.

To recreate the anterior axillary fold, the portion of the graft crossing the axillary crease was tubularized to increase local volume (Fig. 2). Through a separate incision over the prior implant site, a submuscular pocket was developed to accommodate the bulkier medial end of the allograft. This portion was fanned out and sutured to the implant capsule using high-strength sutures, with careful attention to avoid SI rupture. Medial fixation was completed with the shoulder in abduction and external rotation to account for dynamic forces and preserve postoperative range of motion. Both wounds were copiously irrigated and closed in layers. Total operative time was approximately 90 min. A precise institutional acquisition cost for the allograft was not available; however, Achilles tendon allografts are widely used in routine orthopedic procedures, and this technique avoids the additional operative time and donor-site morbidity associated with autologous flap harvest.

## Postoperative course

**Fig. 3:**
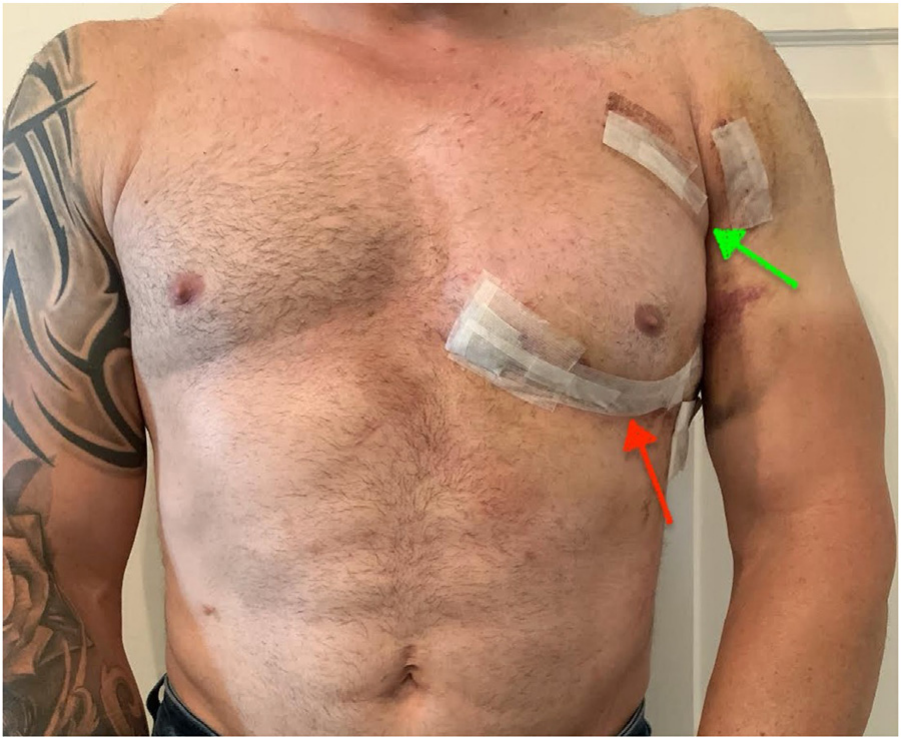
Early postoperative photograph demonstrating wound dressings, expected post-procedural inflammatory changes, restored anterior axillary fold fullness (green arrow), and superior repositioning of the silicone implant (red arrow). Written patient consent for publication of this image was obtained.

**Fig. 4:**
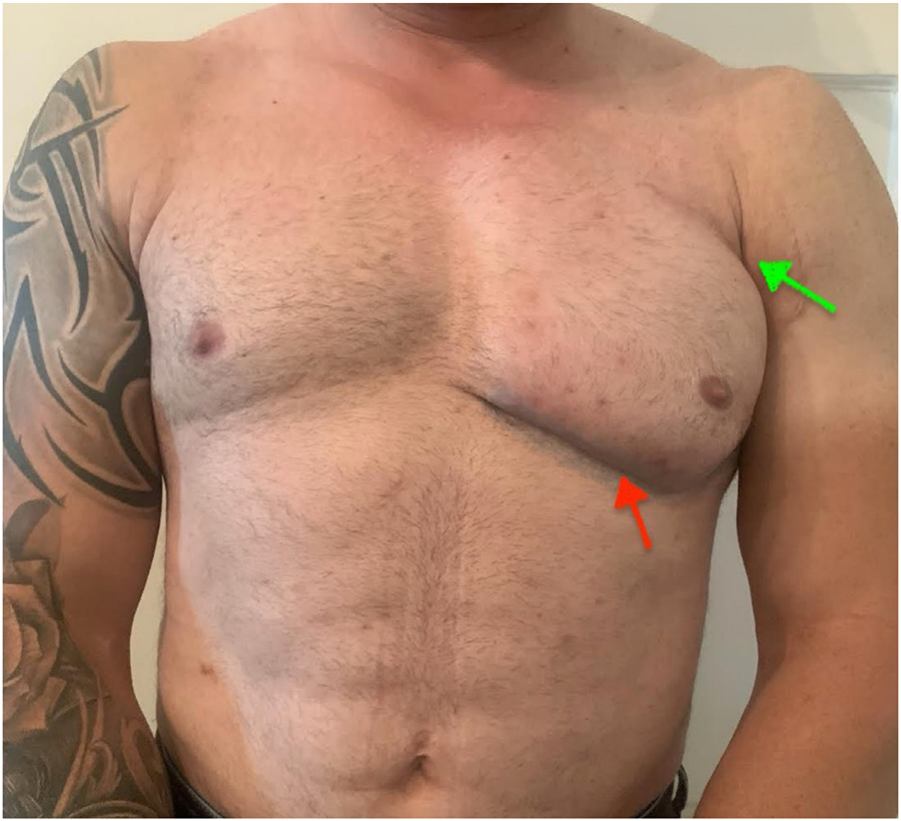
Four-year postoperative result demonstrating durable anterior axillary fold fullness (green arrow) and stable implant position (red arrow). Written patient consent for publication of this image was obtained.

The patient was immobilized in a sling for three weeks, with active range of motion initiated thereafter. Early follow-up demonstrated stable axillary fold reconstruction and appropriate implant positioning (Fig. 3). At three months, he regained full shoulder motion with no functional limitations and no animation deformity during abduction or external rotation. Surgical access was achieved through two incisions: a modified deltopectoral incision (approximately 5 cm) over the distal pectoralis footprint and a separate incision (approximately 5 cm) over the prior implant site. At the four-year evaluation, both incisions had healed as well-matured, flat, non-hypertrophic scars with acceptable color match to the surrounding skin and minimal visibility (Fig. 4). No hypertrophic scarring, keloid formation, widening, or scar-related discomfort was reported, and the patient expressed satisfaction with the scar appearance. At four-year follow-up, the patient reported sustained cosmetic satisfaction, with durable fold contour and no complications, graft failure, or implant displacement (Fig. 4). Evaluation was conducted through serial clinical examinations, standardized photographs, and patient-reported satisfaction assessment. This study was conducted with institutional review board approval (IRB No 2503,247,239/03.12.2025).

## Discussion

This case describes the first reported use of an Achilles tendon allograft for anterior axillary fold reconstruction in PS. The technique applies tendon-osseous repair principles established in pectoralis major rupture surgery^7^^,^^8^ to a distinct reconstructive challenge. The allograft's natural tubular morphology is particularly suited to this application, allowing tubularization at the axillary crease to simulate the volume and projection of a native pectoralis major muscle belly without requiring autologous tissue harvest.

Compared to LD flap transfer, this approach avoids donor-site shoulder morbidity, including adduction and extension weakness reported in long-term follow-up studies.^4^ It also eliminates a separate axillary or back incision, reducing overall surgical burden. Compared to fat grafting, the structural allograft provides more predictable and durable volume without resorption, a recognized limitation of lipofilling in chest wall reconstruction.^6^ Integration with the existing implant capsule allowed contour restoration without implant exchange, offering a less invasive revision strategy.

As with any allograft-based reconstruction, several potential complications warrant consideration. These include graft failure or attenuation over time, suture-anchor pull-out, surgical-site infection, wound-healing complications, a theoretical risk of donor disease transmission (now exceedingly rare with contemporary donor screening and processing), and a theoretical immunogenic response to the graft. Reported complication rates for Achilles tendon allografts in established orthopedic applications, including anterior cruciate ligament reconstruction and pectoralis major and extensor mechanism repair, are low,^7^, ^8^, ^9^ consistent with the uneventful course observed here. In the present case, no graft failure, infection, implant displacement, or animation deformity occurred across four years of follow-up. The single-case nature of this report nonetheless precludes meaningful estimation of complication rates for this specific indication.

From a biological standpoint, Achilles tendon allografts have a well-established safety and integration profile in orthopedic applications, including anterior cruciate ligament reconstruction and extensor mechanism repair. Following implantation, the acellular collagen scaffold undergoes a well-described process of graft incorporation: progressive revascularization and repopulation by host fibroblasts, analogous to the “ligamentization” described after allograft ACL reconstruction, with gradual remodeling toward native tendon architecture.^10^ This biological incorporation is thought to underlie the long-term structural stability seen in tendon allograft applications. With respect to infection, modern allograft processing, comprising rigorous donor screening, aseptic recovery, and terminal sterilization, has reduced allograft-associated infection rates to very low levels,^11^ and the absence of a vascularized autologous donor site may further limit certain wound-healing and infectious complications. Although revascularization and host-cell repopulation were not directly assessed histologically or radiographically in the present case, the durable clinical result at four years, without graft failure or displacement, is consistent with successful biological integration.

Concern regarding implant animation was specifically monitored throughout follow-up. Medial fixation with the shoulder in abduction and external rotation was used to account for dynamic forces during the procedure. No displacement or animation deformity was observed over four years of serial examination, supporting the biomechanical adequacy of capsule-based fixation.

Limitations include the single-case nature of this report, reliance on subjective aesthetic assessment and patient-reported satisfaction, and the absence of objective functional outcome instruments. Generalizability to female PS patients or those without prior SI placement remains to be established. Larger comparative series with standardized outcome measures are needed to confirm reproducibility and durability relative to established techniques. In addition, photographic documentation was obtained as part of routine clinical care rather than under standardized photographic conditions, and aesthetic outcomes were not graded with a validated instrument; prospective standardized imaging and a validated scar-assessment tool (e.g., the Patient and Observer Scar Assessment Scale) would strengthen aesthetic outcome reporting in future series.

## Conclusion

Achilles tendon allograft implantation successfully restored the anterior axillary fold in a PS patient with prior SI placement, with durable results and no complications at four years. This technique may serve as a feasible, less morbid adjunct to conventional reconstruction, warranting validation through larger prospective studies.

## Ethics statement

This study was conducted with institutional review board approval (IRB No 2503,247,239/03.12.2025). Written informed consent for surgery and use of clinical images was obtained from the patient. All identifying features not considered relevant to the reported findings have been obscured in the accompanying figures.

## Financial disclosure

No financial support was received from any organization for the submitted work. The authors have no financial relationships with any organizations that might have an interest in this work.

## Declaration of competing interest

The authors report no competing interests.
